# Changes in Muscle Stiffness in Infants with Congenital Muscular Torticollis

**DOI:** 10.3390/diagnostics9040158

**Published:** 2019-10-23

**Authors:** Dongmin Hwang, Young Ju Shin, Ja Young Choi, Soo Jin Jung, Shin-seung Yang

**Affiliations:** 1 Department of Rehabilitation Medicine, College of Medicine, Chungnam National University, Daejeon 35015, Korea; dmcddm@cnuh.co.kr (D.H.); elarien@cnuh.co.kr (Y.J.S.); jaychoi3399@gmail.com (J.Y.C.); 2 Daejeon Chungcheong Regional Medical Rehabilitation Center, Chungnam National University Hospital, Daejeon 35015, Korea; 3 Department of Rehabilitation Medicine, Hallym University Medical, Center Dongtan Hospital, Hwaseong 18450, Korea; werch@hallym.or.kr

**Keywords:** congenital muscular torticollis, ultrasonography, acoustic radiation force impulse, shear wave velocity, cervical range of motion

## Abstract

Congenital muscular torticollis (CMT) results from unilateral shortening of the sternocleidomastoid (SCM) muscle, usually associated with a fibrotic mass. Although CMT may resolve with physical therapy, some cases persist, resulting in long-term musculoskeletal problems. It is therefore helpful to be able to monitor and predict the outcomes of physical therapy. Shear-wave velocity (SWV) determined by acoustic radiation force impulse (ARFI) elastography can provide a quantitative measure of muscle stiffness. We therefore measured SCM SWV in 22 infants with unilateral CMT before and after 3 months of physical therapy and evaluated the relationships between SWV and SCM thickness and various clinical features, including cervical range of motion (ROM). SWV was initially higher and the ROM was smaller in affected muscles before physical therapy. SWV decreased significantly (2.33 ± 0.47 to 1.56 ± 0.63 m/s, *p* < 0.001), indicating reduced stiffness, and muscle thickness also decreased after physical therapy (15.64 ± 5.24 to 11.36 ± 5.71 mm, *p* < 0.001), both in line with increased neck ROM of rotation (64.77 ± 18.87 to 87.27 ± 6.31°, *p* < 0.001) and lateral flexion (37.50 ± 11.31 to 53.64 ± 9.41°, *p* < 0.001). However, the improved ROM more closely reflected the changes in SWV than in muscle thickness. These results suggest that a change in SWV detected by ARFI elastography could help to predict improvements in clinical outcomes, such as stiffness-related loss of motion, in patients with CMT undergoing physical therapy.

## 1. Introduction

Congenital muscular torticollis (CMT) is a common muscular disorder occurring at or shortly after birth as a result of unilateral shortening of the sternocleidomastoid (SCM) muscle [1]. Shortening of the SCM causes clinical symptoms including head tilt toward the ipsilateral side and chin rotation to the opposite side [2]. Long-lasting severe CMT can result in asymmetric facial structures and cranial bone and cervical spine dysmorphism [3]. The prevalence of CMT in newborn infants or young children is reported to be about 0.3–2% [4]. Various causes of CMT have been reported, including traumatic delivery, primary myopathy, fibrosis due to peripartum bleeding, intrauterine postural abnormality, and intrauterine or perinatal compartment syndrome theory and the hereditary hypothesis; however, the exact etiology of CMT remains unclear [1,4,5].

Macdonald et al. classified SCM patients as having either fibromatosis colli with palpable mass or idiopathic muscular torticollis with shortening of the SCM without a mass [6,7]. Fibromatosis colli occurs in about 0.3–2% of live births [2]. The palpable mass usually grows for up to 1–2 months after birth and then gradually decreases in size and disappears by the second year [8]. However, about 20% of patients have a persistent mass and may require surgical treatment [9]. This abnormality is caused by endomysial fibrosis with collagen deposition, and migration of fibroblasts to the individual muscle fibers undergoing atrophy [10]. Muscle fibrosis can reduce the elasticity of the muscles, thereby limiting the range of neck motion and ultimately reducing muscle function and inducing contracture.

Ultrasonography is an effective tool for evaluating fibrotic lesions within the SCM and for measuring muscle or mass thickness and is therefore widely used in musculoskeletal medicine [11,12]. Ultrasound elastography has been used as a noninvasive method for evaluating and detecting tumors in various tissues, including the liver, breast, kidney, thyroid, and spleen, and has also been developed to allow the objective assessment of muscle stiffness [13,14,15,16]. Acoustic radiation force impulse (ARFI) elastography is an ultrasound elastography technique in which stress or force is applied to deform the target tissue, and the tissue’s mechanical properties are then evaluated by detecting this internal physiological motion. In ARFI, stress is applied by a localized impulsive radiation force generated by a single ultrasound transducer, avoiding the need for the sonographer to compress the tissue mechanically, using a probe for tissue displacement [17]. Tissue stiffness is measured by the speed of shear-wave propagation away from the radiation force region of excitation, with a higher shear-wave velocity (SWV) indicating greater tissue stiffness [15].

We hypothesized that quantitative measurement of the stiffness of the SCM could improve our understanding of the status of CMT patients and help to predict their prognosis. We therefore aimed to investigate the stiffness of the SCM using elastography with ARFI imaging to determine the relationship between the severity of muscle stiffness and clinical features such as range of motion (ROM) in infants with CMT. We hypothesized that the SWV value measured by ARFI may be strongly associated with the cervical ROM.

## 2. Materials and Methods

### 2.1. Subjects

Twenty-two infants (14 boys, 8 girls) with unilateral neck masses of the SCM who were diagnosed with CMT from August 2016 to August 2017 were enrolled in this study (Table 1). The inclusion criteria were age ≤ 3 months with a unilateral SCM mass confirmed by ultrasound examination and diagnosed clinically as CMT by a pediatric rehabilitation specialist. Infants with bilateral SCM masses, congenital anomalies of the cervical spine, spasmodic torticollis, ocular torticollis, or any specific anomaly that could affect the ROM were excluded from this study. Fourteen infants had a mass on the right SCM and eight had a mass on the left SCM. Infants with CMT received physical therapy for 3 months after the first ultrasonographic evaluation including ARFI elastography. This study was conducted according to the principles of the Declaration of Helsinki and was approved by the Ethics Committee the Institutional Review Board of Chungnam University Hospital (No. 2016-08-025-002). The parents of infants gave their informed consent for this study.

### 2.2. Outcome Measurement

**Figure 1:**
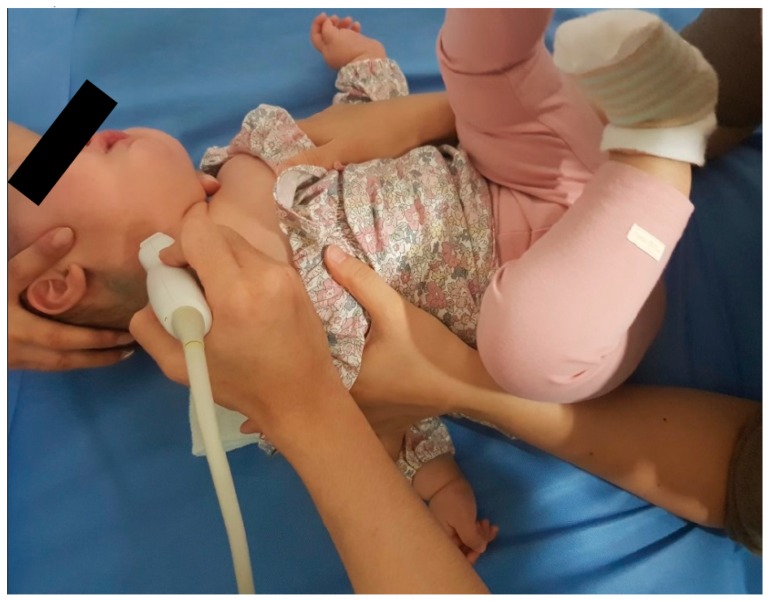
The position of the infant during ultrasonography to the sternocleidomastoid (SCM) muscle in the transverse plane.

**Figure 2:**
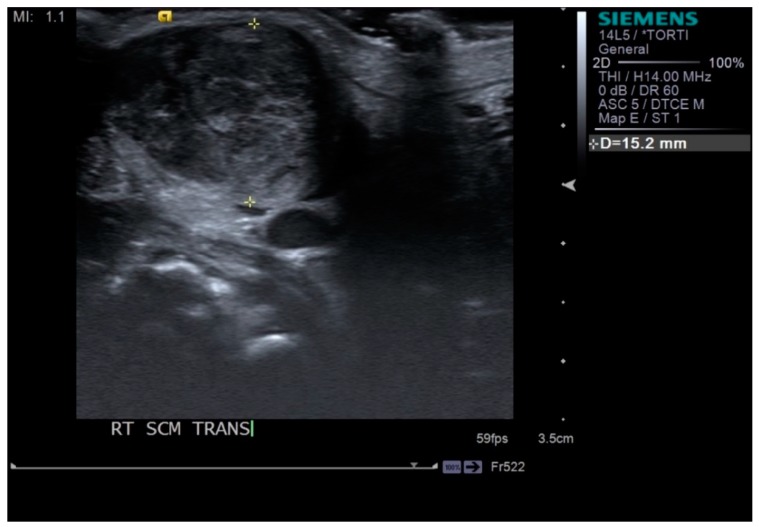
Thickness of sternocleidomastoid (SCM) muscle measured by electronic calipers in the transverse plane.

**Figure 3:**
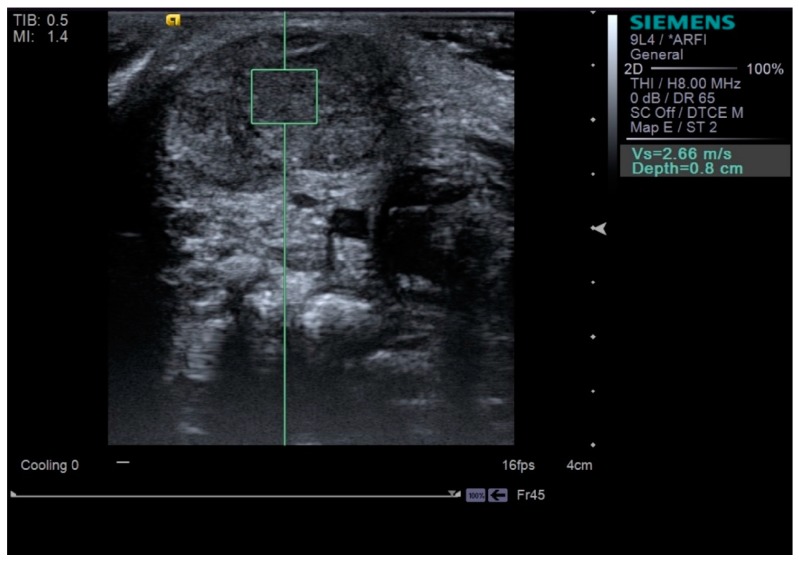
Shear-wave velocity measured by scanning in the transverse plane at the bulbous portion of the sternocleidomastoid muscle (green square means the region of interest).

**Figure 4:**
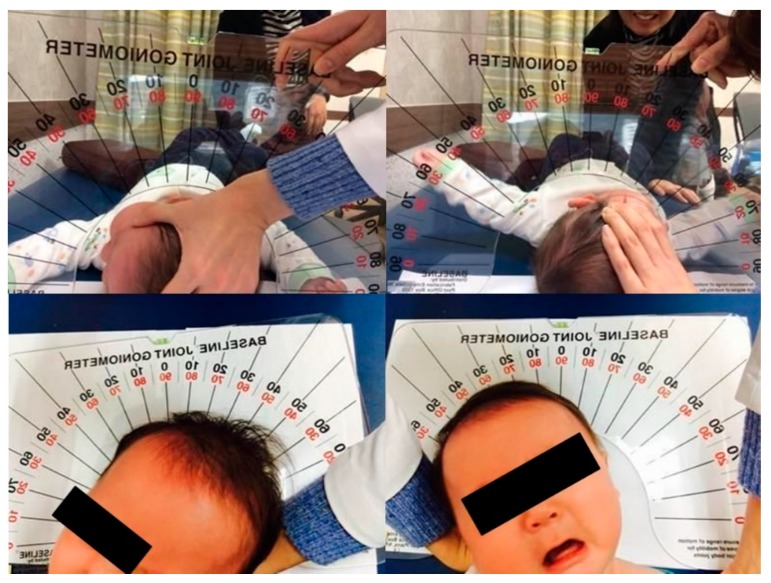
Cervical range of motion measurement using arthrodial goniometer.

Neck ultrasonography was performed while the infants were asleep in a supine position, with slight rotation of the head to the opposite side. A small pillow was used to support the neck, and the infant’s position was also supported by the parents (Figure 1). Scanning was paused temporarily if the infant was uncooperative and cried. The ultrasound procedure was carried out by a skilled physiatrist with > 4 years of experience, using a Virtual Touch Imaging, ACUSON S2000 Ultrasound Unit (Siemens, Mountain View, CA, USA). The thickness of the affected SCM mass was measured in the longitudinal and transverse planes in ultrasound B mode with a linear probe of 5–14 MHz frequency bandwidth (Figure 2). The distance from the superficial to the deep fascia at the thickest portion of the SCM was measured using electronic calipers. The thickness of the unaffected SCM was also measured at the same level as the affected SCM. The SWV of the bilateral SCM was measured using ARFI in the transverse plane (Figure 3). The region of interest was selected to cover the entire SCM. Stronger manual compression increased the speed of shear-wave propagation, and the examiner therefore aimed to apply minimal compression during measurement of the SWV. SWV was measured three times at bilateral SCM muscles, and the median value was used for analysis. The ROM of neck rotation and lateral flexion were measured bilaterally using an arthrodial goniometer (Figure 4).

All infants received physical therapy performed by a skilled pediatric physiotherapist for at least 30 min/week for 3 months. Manual massage and stretching were applied to the affected SCM after applying ultrasound at an intensity of 0.5 W/s for about 5 min. Righting reaction training (if the left side was affected, the patient was leaned to the left side to bring the head upright) was also performed, and parents were instructed to perform the stretching and strengthening exercises at home.

The thickness and SWV of the SCM were remeasured after 3 months of physical therapy, using ultrasonography including elastography and cervical ROM.

### 2.3. Statistical Analysis

Data were analyzed using SPSS for Windows version 22.0 (SPSS Inc., Chicago, IL, USA). Initial ARFI SWV values of the SCM and cervical ROM (neck rotation, lateral flexion) were compared between the affected and unaffected sides using independent *t*-tests. SWV, SCM thickness, and cervical ROM were compared between the initial evaluation and follow-up using paired t-tests and Wilcoxon’s signed rank tests. Pearson’s or Spearman’s correlation analysis were conducted to assess correlations among the initial ARFI SWV, SCM thickness, cervical ROM, and its changes. Statistical significance was set at *p* < 0.05.

## 3. Results

The initial ARFI SWV values, SCM thicknesses, and degrees of cervical rotation and cervical lateral flexion all differed significantly between the affected and unaffected sides. The SWV of the affected side was more similar to that of the unaffected side after 3 months, but the difference was still significant, and the SCM thickness was significantly higher (about twofold) on the affected compared with the unaffected side (Table 2).

The initial SWV value of the SCM showed a strong positive correlation with SCM thickness (r = 0.747, *p* < 0.001) and negative correlations with the degree of cervical rotation and lateral flexion, respectively (r = −0.642 and r = −0.643, both *p* < 0.001) (Table 3).

The ARFI SWV and SCM thickness of the affected side decreased significantly from the initial evaluation to 3 months after the start of the study, while the degree of cervical rotation and lateral flexion increased significantly after 3 months of physical therapy (Table 4).

In addition, the initial SWV value showed a moderate positive correlation with SWV changes (r = 0.518, *p* = 0.014). The change in thickness was also moderately positively correlated with a change in the degree of cervical lateral flexion (r = 0.495, *p* = 0.019). However, there was no significant correlation between the degree of cervical rotation and changes in SWV or thickness of the SCM (Table 5).

## 4. Discussion

The results of the current study showed that the initial ARFI SWV of the affected SCM was faster than that of the unaffected SCM in infants with CMT, and the degrees of neck rotation and lateral flexion were smaller on the affected compared with the unaffected side. Correlation analysis identified a significant positive correlation between the initial SWV and the SCM thickness of both sides, while a faster SWV of the SCM was associated with smaller degrees of cervical rotation and lateral flexion. These findings indicated that the affected SCM was quantitatively less elastic than the unaffected SCM in infants with CMT, and thus hindered free neck motion. Previous studies also demonstrated that SWV could reflect muscle stiffness in patients with spastic cerebral palsy [15,18]. Two studies evaluated the usefulness of sonoelastography in infants with CMT using color-coded real-time sonoelastography [19,20]; however, this technique had limited ability to measure stiffness quantitatively.

ARFI SWV, SCM thickness, and the degrees of cervical rotation and lateral flexion of the affected side all changed significantly between the initial evaluation and after 3 months in patients with CMT. The SWV of the affected SCM was reduced and similar to the unaffected side after 3 months, in accordance with the increasing cervical ROM of the affected SCM, although the SCM remained almost twice as thick on the affected compared with the unaffected side. A previous study also reported that the SWV was significantly higher in patients with severe limitation of motion (LOM) of the neck compared with those with moderate LOM, even though the SCM thicknesses were similar [21]. The mass may increase in size for several weeks and then usually resolves spontaneously by 4–8 months of age, except in about 20% of cases [22]. In this study, the masses were not completely resolved at the 3-month follow-up evaluation, but the SWV was lowered to a value similar to that of the unaffected side. This suggests that a change in the SWV could be an early marker predicting clinical outcome, such as the severity of stiffness of the fibrotic mass, in patients with CMT.

The role of early physical therapy in restoring the cervical ROM has been investigated previously [10,23,24,25], and physical therapy, including stretching, effectively elongated and increased the elasticity of the SCM in CMT infants [23]. However, the effect of physical therapy has not been demonstrated by measuring the correlation between the SWV value and the reduction in stiffness of the SCM. The current results thus provide the first quantitative evidence for a reduction in muscle stiffness after physical therapy in infants with CMT.

The present study found no significant association between the initial ARFI SWV value of the affected SCM and the extent of reduction in SCM thickness or restoration of cervical ROM. This suggests that the initial SWV had a clinically significant association with the initial limitation of neck ROM but did not predict changes in the physical properties of the fibrotic mass after 3 months. We assume that complete restoration of cervical ROM would take longer if the initial SWV of the SCM was fast. However, further studies are needed to investigate the long-term changes in SWV and the fibrotic mass, and their relationship.

Some studies found that the reproducibility of the SWV was greater for scanning in the longitudinal compared with the transverse plane of the muscle axis; however there was no significant difference in SWV value between the two planes [21,26]. Because of the small size of the neck in infants, we therefore measured the SWV of the SCM by scanning in the transverse plane in this study.

This study had several limitations. First, the number of patients was small. Second, there was no control group of infants with CMT who did not receive physical therapy, because of ethical issues involved in not providing physical therapy to patients with LOM of the neck. Third, long-term follow-up was not conducted in this study. Fourth, ultrasonography ARFI was conducted by one physiatrist throughout the study, and it was therefore not possible to assess the inter-rater reliability of the SWV value. But there was intra-operator reliability since the median value was used after measuring 3 times in the SWV.

## 5. Conclusions

The results of the current study indicated that the physical stiffness of the fibrotic mass was reduced and the cervical ROM was restored in infants with CMT after 3 months of physical therapy, despite a still-thickened SCM mass. Assessment of SWV by ARFI may thus provide an alternative method for evaluating SCM stiffness and predicting clinical outcomes in terms of restoring the cervical ROM ahead of changes in muscle thickness in infants with CMT.

## Figures and Tables

**Table 1:** Patient demographics.

Infants with Congenital Muscular Torticollis (*n* = 22)	
Sex (male/female)	14/8
Postpartum age (days)	34.68 ± 19.86
IUP (days)	273 ± 6
Birth weight (g)	3190 ± 262.8
Delivery type (NSVD/C-sec)	15/7
Affected side (right/left)	14/8

Values presented as mean ± standard deviation. Postpartum age = age at time of first evaluation from birth date. IUP = intrauterine pregnancy, NSVD = normal spontaneous vaginal delivery, C-sec = cesarean section.

**Table 2:** Thickness, cervical range of motion, and shear-wave velocity of bilateral sternocleidomastoid muscles comparing the affected and unaffected side at initial evaluation and follow-up.

		Affected Side (*n* = 22)	Unaffected Side (*n* = 22)	*p*-Value
SCM thickness (mm)	Initial	15.64 ± 5.24	5.76 ± 1.62	<0.001 *
	After 3 months	11.36 ± 5.71	6.61 ± 1.06	0.001 *
Cervical rotation ROM (°)	Initial	64.77 ± 18.87	90	<0.001 *
	After 3 months	87.27 ± 6.31	90	0.056
Cervical lateral flexion ROM (°)	Initial	37.50 ± 11.31	57.27 ± 7.67	<0.001 *
	After 3 months	53.64 ± 9.41	57.73 ± 7.52	0.119
SWV of SCM (m/s)	Initial	2.33 ± 0.47	1.25 ± 0.27	<0.001 ^*^
	After 3 months	1.56 ± 0.63	1.22 ± 0.39	0.039 ^*^

Values presented as mean ± standard deviation. SWV = shear-wave velocity, SCM = sternocleidomastoid, ROM = range of motion. * *p* < 0.05 between affected and unaffected sides by independent *t*-test.

**Table 3:** Correlations among initial shear-wave velocity, thickness of sternocleidomastoid muscle, and cervical range of motion.

			Initial SWV	Muscle Thickness
SCM muscle	Initial SWV	Correlation	-	-
		*p*-value		
	Muscle thickness	Correlation	0.747 *	-
		*p*-value	<0.001	
Cervical ROM	Rotation	Correlation	−0.642 *	−0.752 *
		*p*-value	<0.001	<0.001
	Lateral flexion	Correlation	−0.643 *	−0.748 *
		*p*-value	<0.001	<0.001

SWV = shear-wave velocity, SCM = sternocleidomastoid, ROM = range of motion. * *p* < 0.05 in Spearman’s correlation analysis.

**Table 4:** Changes in acoustic-radiation-force-impulse-measured shear-wave velocity of the sternocleidomastoid muscle, sternocleidomastoid muscle thickness, and degrees of cervical rotation and lateral flexion on the affected side before and after physical therapy.

	Initial Evaluation (*n* = 22)	After 3 Months (*n* = 22)	*p*-Value
SCM thickness (mm)	15.64 ± 5.24	11.36 ± 5.71	<0.001 *
Cervical rotation ROM (°)	64.77 ± 18.87	87.27 ± 6.31	<0.001 *
Cervical lateral flexion ROM (°)	37.50 ± 11.31	53.64 ± 9.41	<0.001 *
SWV of SCM (m/s)	2.33 ± 0.47	1.56 ± 0.63	<0.001 *

Values presented as mean ± standard deviation. SWV = shear-wave velocity, SCM = sternocleidomastoid, ROM = range of motion. * *p* < 0.05, paired t-test (SWV of SCM) or Wilcoxon’s signed rank test (SCM thickness, cervical ROM) between initial evaluation and after about 3 months of physical therapy.

**Table 5:** Correlations among initial shear-wave velocity of the sternocleidomastoid muscle and changes in shear-wave velocity, sternocleidomastoid muscle thickness, and cervical range of motion.

			Initial SWV	SWV Change	Thickness Change
SCM muscle	Initial SWV	Correlation	-	-	-
		*p*-value			
	SWV changes	Correlation	0.518 *	-	-
		*p*-value	0.014		
	Thickness changes	Correlation	0.181	0.151	-
		*p*-value	0.420	0.502	
Cervical ROM	Rotation changes	Correlation	−0.253	−0.073	0.141
		*p*-value	0.255	0.746	0.531
	Lateral flexion changes	Correlation	−0.007	−0.026	0.495 *
		*p*-value	0.974	0.910	0.019

SWV = shear-wave velocity, SCM = sternocleidomastoid, ROM = range of motion. SWV change = initial SWV value—later SWV value. SCM muscle thickness change = initial SCM muscle thickness—later SCM muscle thickness. Cervical ROM change = later ROM—initial ROM. * *p* < 0.05 in Pearson’s correlation analysis.

## References

[1] Lee Y.T., Park J.W., Lim M., Yoon K.J., Kim Y.B., Chung P.W., Park H.J., Lee S.Y. (2016). A clinical comparative study of ultrasound-normal versus ultrasound-abnormal congenital muscular torticollis. Pm. R..

[2] Skelton E., Howlett D. (2014). Fibromatosis colli: The sternocleidomastoid pseudotumour of infancy. J. Paediatr. Child Health.

[3] Sargent B., Kaplan S.L., Coulter C., Baker C. (2019). Congenital muscular torticollis: Bridging the gap between research and clinical practice. Pediatrics.

[4] Nilesh K., Mukherji S. (2013). Congenital muscular torticollis. Ann. Maxillofac. Surg..

[5] Lee S.J., Han J.D., Lee H.B., Hwang J.H., Kim S.Y., Park M.C., Yim S.Y. (2011). Comparison of clinical severity of congenital muscular torticollis based on the method of child birth. Ann. Rehabil. Med..

[6] Han M.-H., Kang J.Y., Do H.J., Park H.S., Noh H.J., Cho Y.-H., Jang D.-H. (2019). Comparison of clinical findings of congenital muscular torticollis between patients with and without sternocleidomastoid lesions as determined by ultrasonography. J. Pediatr. Orthop..

[7] Macdonald D. (1969). Sternomastoid tumour and muscular torticollis. J. Bone Jt. Surg. Br..

[8] Crawford S.C., Harnsberger H.R., Johnson L., Aoki J.R., Giley J. (1988). Fibromatosis colli of infancy: CT and sonographic findings. Ajr. Am. J. Roentgenol..

[9] Lowry K.C., Estroff J.A., Rahbar R. (2010). The presentation and management of fibromatosis colli. Ear Nose Throat J..

[10] Demirbilek S., Atayurt H.F. (1999). Congenital muscular torticollis and sternomastoid tumor: Results of nonoperative treatment. J. Pediatr. Surg..

[11] Chang K.V., Wu W.T., Huang K.C., Jan W.H., Han D.S. (2018). Limb muscle quality and quantity in elderly adults with dynapenia but not sarcopenia: An ultrasound imaging study. Exp. Gerontol..

[12] Chang K.V., Wu W.T., Han D.S., Ozcakar L. (2017). Static and Dynamic Shoulder Imaging to Predict Initial Effectiveness and Recurrence After Ultrasound-Guided Subacromial Corticosteroid Injections. Arch. Phys. Med. Rehabil..

[13] Boyaci A., Tutoglu A., Boyaci N., Koca I., Calik M., Sakalar A., Kilicaslan N. (2014). Changes in spastic muscle stiffness after botulinum toxin A injections as part of rehabilitation therapy in patients with spastic cerebral palsy. NeuroRehabilitation.

[14] Jeon M., Youn K., Yang S. (2018). Reliability and quantification of gastrocnemius elasticity at relaxing and at submaximal contracted condition. Med. Ultrason.

[15] Bilgici M.C., Bekci T., Ulus Y., Ozyurek H., Aydin O.F., Tomak L., Selcuk M.B. (2018). Quantitative assessment of muscular stiffness in children with cerebral palsy using acoustic radiation force impulse (ARFI) ultrasound elastography. J. Med. Ultrason (2001).

[16] Wan J., Wu R., Yao M., Xu G., Liu H., Pu H., Xiang L., Zhang S. (2018). Acoustic radiation force impulse elastography in evaluation of triple-negative breast cancer: A preliminary experience. Clin. Hemorheol. Microcirc..

[17] Brandenburg J.E., Eby S.F., Song P., Zhao H., Brault J.S., Chen S., An K.N. (2014). Ultrasound elastography: The new frontier in direct measurement of muscle stiffness. Arch. Phys. Med. Rehabil..

[18] Bilgici M.C., Bekci T., Ulus Y., Bilgici A., Tomak L., Selcuk M.B. (2018). Quantitative assessment of muscle stiffness with acoustic radiation force impulse elastography after botulinum toxin A injection in children with cerebral palsy. J. Med. Ultrason (2001).

[19] Kwon D.R., Park G.Y. (2012). Diagnostic value of real-time sonoelastography in congenital muscular torticollis. J. Ultras Med..

[20] Lee S.Y., Park H.J., Choi Y.J., Choi S.H., Kook S.H., Rho M.H., Chung E.C. (2013). Value of adding sonoelastography to conventional ultrasound in patients with congenital muscular torticollis. Pediatr. Radiol..

[21] Park G.Y., Kwon D.R., Kwon D.G. (2018). Shear wave sonoelastography in infants with congenital muscular torticollis. Med. (Baltim.).

[22] Adamoli P., Pavone P., Falsaperla R., Longo R., Vitaliti G., Andaloro C., Agostino S., Cocuzza S. (2014). Rapid spontaneous resolution of fibromatosis colli in a 3-week-old girl. Case Rep. Otolaryngol..

[23] He L., Yan X., Li J., Guan B., Ma L., Chen Y., Mai J., Xu K. (2017). Comparison of 2 dosages of stretching treatment in infants with congenital muscular torticollis: A randomized trial. Am. J. Phys. Med. Rehabil..

[24] Celayir A.C. (2000). Congenital muscular torticollis: Early and intensive treatment is critical. A prospective study. Pediatr Int..

[25] Petronic I., Brdar R., Cirovic D., Nikolic D., Lukac M., Janic D., Pavicevic P., Golubovic Z., Knezevic T. (2010). Congenital muscular torticollis in children: Distribution, treatment duration and out come. Eur. J. Phys. Rehabil. Med..

[26] Cortez C.D., Hermitte L., Ramain A., Mesmann C., Lefort T., Pialat J.B. (2016). Ultrasound shear wave velocity in skeletal muscle: A reproducibility study. Diagn. Interv. Imaging.

